# Barriers and Facilitators of NHS Health Checks in Socioeconomically Deprived Communities in the North East of England: A Qualitative Study With Peer Researchers

**DOI:** 10.1111/hex.70199

**Published:** 2025-04-15

**Authors:** Judith Eberhardt, Laura Kane, Robert Portman, Jonathan Ling, Tracy Goddard, Mark Johnston, Claire Robinson, Abigail Reay, Andrew Divers, Dorothy Newbury‐Birch

**Affiliations:** ^1^ School of Social Sciences, Humanities and Law Teesside University UK; ^2^ Independent Researcher Darlington UK; ^3^ Public Health Directorate Hartlepool Borough Council Hartlepool UK

**Keywords:** barriers, facilitators, NHS Health Checks, peer researchers, socioeconomic deprivation

## Abstract

**Introduction:**

Preventive health services, such as the NHS Health Check programme, aim to identify and address key health risks, yet participation is particularly low in socioeconomically deprived areas, such as the North East of England. Understanding barriers and facilitators to engagement is critical to improving access and outcomes for these communities. This study aimed to explore barriers and facilitators to NHS Health Check attendance in these underserved communities using a participatory research approach.

**Methods:**

This study employed a qualitative design with a participatory approach, involving peer researchers from the target communities. Two peer research associates (PRAs) from socioeconomically deprived areas were trained to conduct semi‐structured online or telephone interviews with 12 community members eligible for NHS Health Checks. Additionally, 5 stakeholders involved in the programme's delivery were interviewed. Thematic analysis was conducted in collaboration with the PRAs to ensure community perspectives were authentically captured.

**Results:**

Barriers to participation included limited awareness, cultural perceptions of self‐reliance, fear of health‐related discoveries, mistrust of healthcare systems and logistical challenges exacerbated by structural inequalities. Participants emphasised the need for culturally tailored communication and flexible, accessible health checks. Stakeholders highlighted the role of collaboration, targeted outreach and digital tools in addressing these barriers.

**Conclusion:**

The study highlights key barriers to NHS Health Check uptake in socioeconomically deprived communities in the North East of England. Improving communication, increasing accessibility through community‐based services and building trust in healthcare are recommended key strategies to enhance participation and reduce health inequalities in these regions.

**Patient or Public Contribution:**

Peer researchers, individuals with lived experience of being from socioeconomically deprived communities in North East England and eligible for NHS Health Checks, were involved in the design and conduct of this study. They were trained to conduct interviews with community members and contributed to the thematic analysis, ensuring that public perspectives were integral to the interpretation of the data.

## 1 Introduction

Cardiovascular disease (CVD) is one of the leading causes of death and morbidity in the United Kingdom (UK), with approximately 7.6 million people currently living with heart and circulatory diseases. CVD is responsible for 27% of UK deaths each year, accounting for 170,000 lives lost, with nearly 49,000 of these occurring before the age of 75 [1]. The North East of England experiences disproportionately high rates of premature mortality due to CVD [2]. These elevated rates are driven by a combination of socioeconomic deprivation, higher prevalence of lifestyle‐related risk factors and inequitable access to preventive health services [1].

There is a strong link between socioeconomic deprivation and health inequalities, particularly in relation to CVD outcomes. Residents of the UK's most deprived areas are nearly four times more likely to die prematurely from CVD than those in more affluent areas [3, 4]. This risk is further compounded by other factors, such as ethnicity, age and comorbid conditions. CVD prevalence is higher among males, older adults, those with severe mental illness and individuals from South Asian or African Caribbean backgrounds [3, 5]. These groups are often less likely to engage with preventive health services, creating a pressing need for targeted, accessible interventions.

In addition to the human cost, the financial impact of CVD on the NHS and the UK economy is significant, with an estimated annual cost of £25 billion. Importantly, many of the risk factors for CVD are modifiable. Between 50% and 80% of CVD cases are preventable through interventions targeting behaviours such as smoking, excessive alcohol consumption, physical inactivity, poor diet and the management of conditions like high blood pressure and cholesterol [6, 7]. These modifiable risk factors tend to be more prevalent in socioeconomically deprived communities, where individuals are often disproportionately affected by CVD and related conditions such as type 2 diabetes, kidney disease and stroke [6] and face structural and social determinants that compound health risks. For example, financial constraints may limit access to nutritious food and opportunities for physical activity, while stress associated with economic instability can contribute to higher rates of smoking and alcohol consumption [8, 9, 10]. Additionally, environmental factors, such as living in areas with fewer green spaces or higher pollution levels, can exacerbate these risks [9, 10]. Deprivation also often intersects with limited access to healthcare services and preventive interventions, further exacerbating health inequalities [9, 10].

Cultural norms and community‐specific dynamics may further compound these challenges. Socioeconomically deprived populations often face entrenched barriers linked to systemic inequities, including limited social capital, power imbalances and constrained access to resources. These factors influence behaviours such as engagement with preventive health services [11], highlighting the need for culturally informed interventions.

The National Health Service (NHS) Health Check programme, launched in 2009, represents a large‐scale public health intervention aimed at addressing modifiable risk factors and reducing health inequalities. The programme is targeted at individuals aged 40–74 who do not have a pre‐existing diagnosis of CVD. It aims to prevent cardiovascular events through early detection of risk factors and lifestyle interventions. Through a combination of biological testing and lifestyle assessments, the programme evaluates the risk of developing CVD, stroke, type 2 diabetes, or kidney disease over the next 10 years [12]. Those found to be at increased risk are referred to relevant lifestyle services or for further medical follow‐up, providing an opportunity to reduce future CVD incidence and healthcare costs.

Evidence indicates that NHS Health Checks are effective in identifying undiagnosed conditions, such as hypertension and diabetes, enabling earlier management and reducing long‐term health risks [13]. Additionally, the programme has been shown to be cost‐effective, particularly when implemented in socioeconomically deprived communities, where the burden of preventable conditions is greatest [14].

Uptake of the NHS Health Check programme remains inequitable, particularly among individuals from socioeconomically deprived communities, where its impact could be greatest. Women, older individuals and those from higher socioeconomic backgrounds are more likely to attend, while men, smokers, and those from deprived backgrounds—often at the highest risk for CVD—are less likely to engage [3, 15]. This disparity is especially concerning in regions such as the North East of England, which experiences significant health inequalities, including lower life expectancy, and premature mortality from CVD compared to national averages [2, 16].

Efforts to enhance NHS Health Check uptake in the North East have included piloting the service in community pharmacies and other nontraditional healthcare settings, aiming to reach populations that face greater barriers to access. However, these initiatives have faced challenges such as technological barriers and inconsistent service delivery across different areas [17]. Given the complexity of increasing health check engagement across diverse community settings, tailored approaches that consider local contexts and barriers are necessary.

To help address these challenges, this study aimed to explore the barriers and facilitators to NHS Health Check participation among individuals living in socioeconomically deprived areas of the North East of England. The study took a participatory research approach, a valuable tool for public health research [18, 19, 20], involving community members as peer research associates (PRAs) to capture a more detailed understanding of the local context. These PRAs were individuals from the target population who had lived experience of the challenges associated with accessing healthcare. Their involvement in this study ensured that the voices of those most affected by health inequalities were central to the research process. In addition, the study involved key stakeholders from local public health and healthcare organisations, whose perspectives were essential for understanding the broader structural challenges facing the NHS Health Check programme. Through co‐producing research with community members and embedding their perspectives in the analysis, this study aimed to inform strategies for enhancing NHS Health Check engagement and ultimately reducing health inequalities in the region.

## 2 Methods

### 2.1 Design

This study employed a qualitative research design to explore the barriers and facilitators associated with attending NHS Health Checks in socioeconomically deprived areas of the North East of England. Semi‐structured interviews were conducted with community members living in deprived areas and eligible for NHS Health Checks, and with stakeholders involved in the programme's delivery. The interviews aimed to gain an in‐depth understanding of the experiences, perceptions and barriers faced by community members in accessing health checks, as well as stakeholders' perspectives on the programme's implementation and areas for improvement.

### 2.2 Research Team

A participatory research approach was adopted to ensure that the voices and lived experiences of community members were central to the study. PRAs, who were individuals from underserved communities and themselves eligible for health checks, were employed to conduct interviews. This approach fostered collaboration and coproduction, enhancing the relevance and impact of the findings.

These individuals were selected based on their shared experiences with the target population and underwent training in ethical interviewing, confidentiality and maintaining neutrality in discussions. Before conducting interviews independently, the PRAs observed one interview conducted by a research associate to understand the expected approach. Each PRA‐led interview was observed by an academic researcher and was followed to ensure quality and rigour in data collection. The PRAs were compensated in the form of shopping vouchers for their participation in the training and for conducting interviews. The vouchers were provided in accordance with the lead institute's policy. Themes were co‐developed with PRAs during the data analysis phase to ensure that the analysis reflected community perspectives authentically. Both PRAs were offered co‐authorship on this manuscript to acknowledge their contributions.

Furthermore, a steering group, consisting of four representatives from local authorities across the North East of England and one regional Office for Health Improvement and Disparities (OHID) Health Check representative, was established to oversee the research. The steering group provided feedback on the interview schedules and supported recruitment efforts by disseminating information about the study through their networks. Their involvement helped to ensure that the study remained relevant to the needs of local stakeholders and that the findings would have practical implications for the delivery of NHS Health Checks in the region.

### 2.3 Participant Recruitment

#### 2.3.1 Community Members

Eligible participants were adults aged 40–74 years living in socioeconomically deprived areas of the North East of England, as defined by Index of Multiple Deprivation (IMD) scores between 1 and 3 [21]. Participants also needed to have no prior history of CVD and be eligible for an NHS Health Check. Both individuals who had and had not accessed an NHS Health Check were included to provide a comprehensive view of the barriers and facilitators to participation.

Recruitment of community members employed a multifaceted approach to maximise participation. Sign‐up links for the study were distributed via social media and professional networks, including community organisations, local councils, public health professionals and educational institutions. Additionally, posters and online posts were used. During screening, participants provided their postcodes to confirm residence in eligible areas. Shopping vouchers for participation were sent to home addresses, serving as an additional validation step.

#### 2.3.2 Stakeholders

Stakeholders were recruited based on their professional involvement in the NHS Health Check programme. These individuals provided insights into the programme's implementation, including barriers, facilitators and potential improvements, particularly for underserved communities. Stakeholders included local public health professionals, representatives from local councils, and other relevant organisations. Due to ethical constraints, individuals directly employed by the NHS were excluded from participation.

Stakeholder recruitment was facilitated by gatekeepers who ensured relevance to the study. Recruitment efforts included targeted outreach to professional networks and collaborations with public health organisations.

### 2.4 Materials

The interview schedule for community members explored their perceptions, experiences and barriers related to NHS Health Checks (see Table S1). It included questions designed to elicit information on participants' awareness and understanding of the programme, their experiences of attending (or not attending) a health check, and their suggestions for improving the service. The schedule also included demographic questions to ensure eligibility, including age, gender, ethnicity, education level and postcode.

Stakeholders were interviewed using a schedule that explored their perspectives on the NHS Health Check programme (see Table S2), focusing on barriers and facilitators, particularly in relation to engaging underserved communities. Stakeholders were also asked to provide their views on the effectiveness of current strategies, potential improvements and the role of technology and innovation in enhancing the programme's reach. The interview concluded by asking about any additional thoughts or recommendations for the future of the programme.

### 2.5 Procedure

Data were collected between May and August 2024, following ethics approval from Teesside University's Research Ethics Humanities and Social Sciences Sub‐Comittee (Review Reference 2024 May 21984 Eberhardt). Interviews with community members were conducted either online via Microsoft Teams or by telephone, depending on participant preference. Each interview lasted approximately 30–45 minutes and was conducted by either a research associate or the PRAs. Participants were briefed on their rights, including confidentiality, the right to withdraw, and the option to pause or stop the interview at any time. Stakeholder interviews were also conducted online via Microsoft Teams and lasted 30–60 minutes.

### 2.6 Analysis

Thematic analysis [22] was used to analyse the qualitative data from both community members and stakeholders. The analysis process began with J.E. familiarising themselves with the data by reading and rereading the interview transcripts to gain a deep understanding of the content. Following this, codes were identified inductively by J.E., i.e., developed directly from the data rather than being predetermined. These codes represented key concepts, ideas, or recurring patterns within the data.

Once initial coding was completed, the codes were systematically applied to all transcripts by J.E., and then grouped into broader categories based on their conceptual similarity. These categories were refined through discussions with L.K., M.J. and T.G., ensuring agreement on how the data should be organised. From these categories, themes were developed through an iterative process of review and refinement. This involved identifying overarching patterns or narratives that encapsulated the main findings and organising the themes hierarchically where necessary, such as including subthemes. Collaboration with the PRAs, M.J. and T.G., was central to this process, to ensure the themes reflected the perspectives of the community members. This co‐production approach allowed for the integration of lived experiences and local knowledge into the analysis. The final themes were reviewed, named and written up in alignment with the Standards for Reporting Qualitative Research (SRQR) [23].

Data from community members and stakeholders were analysed separately, to ensure that the distinct perspectives of these groups were fully recognised. Community members shared experiences shaped by their personal interactions with healthcare services, while stakeholders offered insights informed by their professional responsibilities and system‐level understanding. Separating these perspectives allowed for a focused exploration of both individual and structural barriers, ensuring that the analysis remained nuanced and meaningful. This approach was taken to provide insights that address challenges at both the community and policy levels.

### 2.7 Reflexivity

The research team actively reflected on their positionality throughout the study to mitigate biases that might arise from their roles and backgrounds. For example, the academic researchers recognised their potential power dynamics when engaging with PRAs and participants and worked to promote an equitable environment by involving PRAs in co‐developing themes during the data analysis phase. Additionally, discussions within the team ensured that interpretations were grounded in participants' perspectives rather than shaped by researchers' assumptions.

## 3 Results

### 3.1 Interviews With Community Members

Four themes and two subthemes were developed from the 12 interviews (five of these conducted by the PRAs and the remainder by a research associate) with community members. Table 1 shows interviewees' demographic characteristics.

**Table 1:** Demographic characteristics of community members eligible for NHS Health Checks (*n* = 12).

Characteristic	Categories	Count (*n*)
Gender	Female	10
	Male	2
Ethnicity	White British	11
	Black African	1
Attendance	Attended NHS Health Check	5
	Did not attend	7
Age range	40–49	6
	60–69	3
	70+	3

A summary of the themes is displayed in Table 2.

**Table 2:** Themes developed from interviews with community members (*n* = 12).

Theme	Description
1. Lack of awareness and communication gaps	Many participants were unaware of NHS Health Checks or had limited knowledge of their purpose. Those who had attended sometimes found the communication of results unclear or incomplete.
2. Psychological and emotional barriers	
* 2a. Fear of discovering health problems*	Fear of discovering health problems deterred some participants from attending. The thought of confronting potential health issues led to avoidance.
* 2b. Mistrust and stigma in healthcare*	Some participants expressed mistrust toward healthcare professionals. Others spoke of the stigma associated with individuals dealing with addiction, leading them to avoid healthcare.
3. Barriers to access and convenience	Difficulty booking appointments and inconvenient GP surgery locations hindered attendance. Participants suggested more flexible timings and local/community‐based checks.
4. Willingness to attend and suggestions for improvement	Despite barriers, many participants expressed a willingness to attend if invited. Suggestions for improvement included better communication, more flexible appointments and community‐based outreach.

#### 3.1.1 Lack of Awareness and Communication Gaps

Many participants had little or no awareness of the NHS Health Check programme, and those who had heard of it often encountered vague or insufficient information. This lack of clarity about the purpose and benefits of the checks, coupled with inadequate communication from healthcare providers, was a significant barrier. Some participants only became aware of the programme after seeing promotional materials in passing: *‘No [I'd never heard of NHS Health Checks.] Not since I saw your [study information] pop up […] at work’* (Participant 9). Participant 6 mentioned, *‘I didn't know about the NHS Health Check programme before today.] I Googled it to find out what it was […]. And obviously it's something I should know about’.* Some participants expressed confusion about what age the checks begin, how frequently they occur, and how individuals are contacted. This lack of clarity in communication often left participants confused and uncertain about their eligibility or how to engage with the programme. Participant 12 stated, *‘I always believed […] you were called when you turned 40 for the general check over. But it's never happened’.*

The language used to communicate results was often felt to be overly complex and confusing. Participant 2 commented on the letter received from the GP with her results: ‘“*Your blood results from your NHS Health Check are in range […], your risk calculation is 1.4%, which is well within normal range, and then, you know, kind regards” and then who it was from, but you're like, that doesn't really tell me anything’.* Such communication challenges highlight broader issues with the accessibility and user‐friendliness of health information in socioeconomically deprived communities, where individuals may have limited health literacy.

Moreover, some participants highlighted the perception that health checks were just a one‐time event, without any structured follow‐up plan to ensure continuity of care. Participant 5 emphasised the importance of catching problems early: ‘*It's better to know if anything is wrong and, you know, early enough because that's only when you can get help’.* However, Participant 10 raised concerns about the lack of follow‐up, stating, ‘*If they found anything, they would let you know nothing’.* These findings suggest a need for improved communication strategies that clearly explain the purpose of NHS Health Checks and provide consistent follow‐up to maintain trust and engagement.

#### 3.1.2 Fear of Discovering Health Problems

Several participants expressed fear about attending health checks, rooted in the anxiety of potentially discovering health problems. This fear created a barrier, as many felt that learning about an issue would force them to confront difficult choices or lifestyle changes. Some preferred not to know if something was wrong, allowing them to continue their routines without the burden of a diagnosis. This avoidance of bad news was a common deterrent for participants. Participant 8 explained, ‘*I'd rather not know because [of] fear’*. Others echoed similar sentiments, with Participant 4 stating, ‘*Some people don't like to go because they're scared to find out that something is wrong’*.

This anxiety may also reflect the cultural importance of self‐reliance in some working‐class communities, where individuals often prioritise coping independently rather than seeking external support. Such norms can amplify fear and avoidance, as health checks may be seen as challenging one's autonomy or exposing vulnerabilities [24].

#### 3.1.3 Mistrust and Stigma in Healthcare

Several participants described a mistrust of healthcare professionals, which had been exacerbated by previous negative experiences. This mistrust was particularly pronounced among those who felt stigmatised due to conditions such as mental health issues or addiction. Participants shared that this judgement from healthcare providers led to avoidance of preventive services, including NHS Health Checks, as they did not feel comfortable or respected in healthcare settings.

Participant 6 shared, ‘*I know people that have had experience with […] mental health and their GP would have lost a lot of trust and they wouldn't go for kind of physical health stuff’.*

Participant 8, who worked in addiction recovery support, highlighted how stigma sometimes kept individuals from seeking care from their GP: ‘*A lot of our people don't even have a GP. […] So, I think that's a barrier. I think stigma is a barrier as well, for our people, being an addict […] with our presentation and how we come across to the society in the public’.*

The mistrust among participants underlines a broader social dynamic, where marginalised groups may perceive healthcare systems as distant or unaccommodating, which may reflect systemic power imbalances [25].

#### 3.1.4 Barriers to Access and Convenience

Access to NHS Health Checks was sometimes hindered by logistical challenges, particularly in booking appointments. Many participants found it difficult to secure appointments at their GP surgery, often citing long waiting times or the shift to digital systems during the pandemic, which some found inaccessible. For those working nontraditional hours, attending a check during regular working hours was especially challenging: ‘*Trying to get booked in at my GP surgery is a bloody nightmare, to be honest… everything's flaming e‐consult now. […] So, they're just really putting another barrier in’* (Participant 7).

Participant 12 explained:‘*For a lot of people, you know, working shift patterns, you know, working in care or in factories, I think it can probably be quite difficult to get that time to go during the doctor's opening hours’.* The location of health checks also presented an obstacle. Travelling long distances to a GP surgery or another medical facility was not practical for many. Several participants suggested offering health checks in more familiar, accessible community settings.

Participant 12 noted: ‘*GPs do kind of an outreach thing where they could set up in a community centre just to give an opportunity for different places, or even home visits would probably be ideal for some people’.* Participants emphasised the importance of outreach services, especially in the form of informal surgeries in community centres, where NHS Health Checks could be made more accessible. This suggestion was seen as a way of alleviating logistical barriers, particularly for individuals living in more socioeconomically deprived areas or with limited mobility. Additionally, participants reflected on how the challenges of access were compounded by the lack of tailored solutions for working‐class individuals, who often have less flexible work schedules or limited access to transport. This highlights the intersection of logistical barriers with structural inequalities, where systemic challenges disproportionately affect deprived populations [26].

#### 3.1.5 Willingness to Attend and Suggestions for Improvement

Despite various barriers, many participants indicated that they would attend an NHS Health Check if they received a formal invitation. They recognised the value of early diagnosis and preventive care, but for some, a lack of clear communication or accessibility issues had previously deterred them. Participants suggested that improvements in flexibility and communication could significantly increase uptake.

Participant 2 noted that it would depend on symptoms being present, stating, ‘*I would encourage somebody to go [if there were] an early indication that something's not quite right’*. Some participants suggested more flexible appointment times or offering NHS Health Checks in community locations to make attendance easier: ‘*The employer has to give time off to attend these health checks. […] Paid time off would be really great and would probably encourage people. But, you know, just to say that the employer has to be flexible […] to allow for this appointment’* (Participant 12).

Improved communication was another common suggestion, with the same participant recommending ‘*[A] basic leaflet that explains what's going to happen on the day and the benefits of attending this to you in the long term’*. Some participants also suggested that promoting NHS Health Checks through public spaces could raise awareness and encourage more people to attend*: ‘As well as, like, your local corner shop, I think schools would be a good place ‘cause, how many grandparents do the childcare these days; in a window at school grandparents [are] going to see it. And […] as we know, our grandparents are a lot younger these days’* (Participant 9).

Others suggested using social media to raise awareness of NHS Health Checks: ‘*Everyone's got a mobile phone now, you know, it's [the] digital age, isn't it? […] Social media, everyone's on TikTok or something now, you know. […] It's the way to reach out, isn't it? And, like, widen your audience rather than just the traditional ways that worked in the past’* (Participant 4).

Participants thus highlighted the importance of trust and cultural sensitivity in outreach efforts to build stronger connections with individuals who might otherwise remain sceptical about the benefits of health checks. These approaches were seen as ways of enhancing awareness and trust in the healthcare system.

### 3.2 Interviews With Stakeholders

All five stakeholders who were interviewed worked for various councils in the North East of England; the councils they worked for have not been reported to preserve participants' anonymity. Two of the interviews were conducted by the PRAs (M.J. and T.G.), and the remainder by a research associate (L.K.). Table 3 displays the interviewees' roles, and Table 4 summarises the themes generated from these interviews.

**Table 3:** Stakeholder roles.

Stakeholder (SH)	Role
SH1	Public Health Practitioner
SH2	Public Health Team Member (commissioning of NHS Health Checks)
SH3	Clinical Public Health Lead for a local council (commissioning of primary care services)
SH4	Public Health Portfolio Lead
SH5	Office of Health Improvement and Disparities Consultant in Public Health

**Table 4:** Themes from interviews with stakeholders.

Theme	Description
1. Limitations in awareness and access	Stakeholders described the low public awareness of NHS Health Checks and difficulties navigating healthcare systems. These challenges were compounded by inadequate outreach to underserved populations and structural barriers.
2. Challenges in service delivery	Issues were highlighted regarding funding constraints, resource allocation inefficiencies, and reliance on GP practices. Stakeholders expressed concern about the current service model's limitations in reaching priority populations.
3. Innovations and improvements	Stakeholders suggested integrating services across sectors, promoting collaboration and introducing digital and community‐based delivery models. Strategic reforms and innovative approaches were viewed as essential to improving the programme.

#### 3.2.1 Limitations in Awareness and Access

Stakeholders consistently noted that low public awareness NHS Health Checks was a significant barrier to participation. SH5 compared the public's familiarity with cervical screening to the lack of preparedness for health checks: ‘*The public knows about [cervical screening], you know? So [unlike with NHS Health Checks] when you get your invite for your cervical screen, you've already kind of mentally prepared, actually, because you understand what it is’.*

SH3 highlighted how this lack of awareness intersected with other challenges faced by marginalised groups: ‘*I think [health checks are] very targeted towards the ‘worried well’. So, people that have got a priority of their health will go and absolutely have it. But actually, somebody who may be battling abuse or a dependence, their priority is not their health’.*

Structural barriers to accessing services were also emphasised. SH2 commented on difficulties navigating the healthcare system: ‘*A lot of the problems people face with general practice is just about how you navigate the system’.* The current eligibility criteria, such as the age threshold for health checks, were also questioned. SH3 noted: ‘*We are seeing people get poorly earlier, but yet we haven't changed the NHS Health Check from the age of 40. So, there are lots of people… in their 30 s who probably would sit at quite a high [cardiovascular risk score]’.*

Stakeholders also noted that the programme often failed to engage marginalised groups who might not prioritise preventive healthcare. SH3 observed, ‘*I think [health checks are] very targeted towards the “worried well”. So, people that have got a priority of their health will go and absolutely have it. But actually, somebody who may be battling abuse or a dependence, their priority is not their health’*. Stakeholders further noted that awareness campaigns must be culturally appropriate and specific to the needs of different populations. SH1 emphasised the importance of tailoring messages to address both practical barriers and underlying mistrust, stating, ‘*We need to look at involving people or professionals that people from those deprived areas are in contact with or trust already’*.

#### 3.2.2 Challenges in Service Delivery

Stakeholders highlighted significant challenges related to funding and resource allocation. SH5 described the limitations of the public health grant: ‘*The service is funded from the public health grant, and there are well‐documented challenges in terms of how that has grown or not grown over recent times’.* The activity‐based payment system was seen as a further obstacle. SH3 explained, ‘*It's activity‐based, so it's payments by results or payments by activity. And I think that depending on how much you're paid depends on how much a GP wants to take that up’.*

The programme's reliance on GP practices was also identified as a key limitation. SH1 noted, ‘*We need to look at involving people or professionals that people from those deprived areas are in contact with or trust already’.* This model often failed to reach the most at‐risk populations, who might not have regular contact with primary healthcare providers. SH4 remarked on the inefficiencies of the current delivery methods: ‘*I think it does [help] in terms of highlighting issues, hopefully getting things before they get worse in a preventative way, but sometimes obviously it's too late’.*

Several stakeholders pointed out that these challenges were particularly acute in socioeconomically deprived areas, where limited resources and logistical barriers further hindered access to NHS Health Checks.

#### 3.2.3 Innovations and Improvements

Stakeholders proposed a range of strategies to enhance the NHS Health Check programme. Many emphasised the importance of collaboration between local authorities and healthcare providers. SH5 suggested, ‘*We've all got no money, so let's do things together. So, if the NHS are commissioning blood pressure checks in the community, are they not better off using that money with local authorities to scale up the actual NHS Health Check?’*

Digital innovations were viewed as critical for improving accessibility and engagement. SH2 described an initiative where a GP practice integrated health checks into community outreach: ‘*We've got a GP practice that are wanting to do a community engagement event… So, they're taking the GP practice out into the community which is quite a deprived community to promote all the things that general practice offers, but also focusing on offering people a health check’.*

Community‐based models were also seen as essential for addressing logistical barriers and increasing uptake. SH4 commented, ‘*I would prefer if we [were] like other areas and add some community‐based stuff’.* SH3 suggested expanding the scope of NHS Health Checks to include additional health topics, such as menopause, to make the programme more relevant and appealing. Overall, stakeholders agreed that strategic reforms and innovative approaches were necessary to ensure the programme's sustainability and effectiveness.

## 4 Discussion

The present study explored the barriers and facilitators to NHS Health Check attendance among community members living in socioeconomically deprived areas of the North East of England, and stakeholders involved in delivering the programme. The interviews with both groups identified a range of barriers to NHS Health Check attendance. These included limited public awareness and poor communication about the programme, psychological barriers such as fear of diagnosis, mistrust in healthcare professionals due to stigma or past negative experiences, logistical challenges like inconvenient appointment times and inaccessible locations, and systemic issues including underfunding and reliance on GP‐based delivery models. These findings are discussed next.

### 4.1 Public Awareness and Engagement

Limited public awareness emerged as a significant barrier, with participants frequently describing being unaware of the programme or unclear about its purpose. This aligns with findings in the literature that inadequate communication and outreach hinder engagement with preventive health services. Previous studies have demonstrated that centralised and consistent public health campaigns, such as those used for vaccination programmes, significantly improve awareness and uptake [27, 28]. Stakeholders in the present study emphasised the absence of national promotional efforts for NHS Health Checks in recent years, particularly in the context of organisational changes within the NHS. The discontinuation of centralised materials has resulted in gaps in public understanding and preparedness, reducing the effectiveness of local initiatives.

Psychological barriers also played a critical role in engagement, particularly fear of diagnosis. Participants expressed anxiety about discovering health problems, which aligns with research showing that the fear of life‐changing diagnoses, especially for conditions like cardiovascular disease or cancer, discourages individuals from seeking preventive care [29]. This fear can lead to avoidance behaviours, undermining the early detection benefits of health checks.

Trust in healthcare professionals was another influential factor. Participants reported mistrust stemming from previous negative experiences or perceptions of stigma. These findings reflect a broader issue identified in the literature, where individuals from lower socioeconomic backgrounds often avoid healthcare services due to concerns about discrimination [30]. Building trust through community‐based approaches that involve local leaders and peer educators has been shown to address these barriers effectively [31, 32].

The findings highlight disparities in cultural health capital, the knowledge and resources needed to navigate healthcare systems [33]. In deprived communities, limited familiarity with preventive health or low confidence in engaging with healthcare professionals may heighten barriers [25].

### 4.2 Care Delivery and Systemic Challenges

Accessibility and logistical barriers were widely reported by both community members and stakeholders. Participants highlighted issues such as inconvenient appointment times, long waiting periods, and difficulties navigating GP systems, which compounded existing inequities in accessing healthcare. These challenges are consistent with findings that rigid, GP‐based delivery models are insufficient for reaching diverse populations [3, 34]. Prior research further emphasises how systemic inefficiencies, including resource constraints and lack of localised adaptations, can disproportionately hinder healthcare engagement in deprived communities [11].

Stakeholders also emphasised the limitations of the current delivery model, noting that it relies heavily on traditional GP surgeries, which may not be practical or welcoming for many. Suggestions were made to integrate health checks into community settings, such as local libraries, schools, or mobile clinics; these align with evidence supporting the success of community outreach in improving service accessibility [34, 35].

Systemic issues such as inadequate funding and the constraints of an activity‐based payment system were identified as significant obstacles to improving NHS Health Check delivery. Stakeholders noted that the public health grant has not kept pace with increasing demand, limiting the ability to innovate or expand services. The current funding model incentivises outputs rather than outcomes, leading to inconsistencies in service delivery and reduced capacity to address health inequalities effectively. These challenges mirror critiques in the broader literature, which call for a shift towards funding structures that prioritise holistic, patient‐centred care [36].

### 4.3 Opportunities for Strategic Improvement and Innovation

Stakeholders identified opportunities for innovation and collaboration as crucial to addressing these challenges. Digital tools were highlighted as a means to streamline processes, from booking appointments to delivering follow‐up care, with evidence suggesting that such technologies can enhance engagement in preventive programmes [37]. Mobile apps and text message reminders, for example, have been successfully employed in other public health initiatives to improve attendance rates [38]. Stakeholders also proposed expanding the scope of NHS Health Checks to address related health issues, such as menopause, and to incorporate personalised interventions. These suggestions align with calls in the literature to broaden the scope of preventive health services to better meet diverse community needs [39].

Collaboration between local authorities, public health bodies and community organisations was another prominent theme. Stakeholders stressed the importance of unified efforts to address health inequalities, with local partnerships offering a mechanism to leverage community resources and enhance programme delivery. Previous research supports the effectiveness of co‐designed interventions that involve community input, particularly in socioeconomically deprived areas where trust in traditional healthcare systems may be low [35].

### 4.4 Recommendations for Improving NHS Health Check Uptake in Socioeconomically Deprived Communities in North East England

Our findings suggest several practical steps for improving the uptake of NHS Health Checks in socioeconomically deprived communities in North East England. First, raising public awareness is important. Many people remain unaware of the programme; thus, a national campaign, supported by local efforts, could highlight the importance of early detection for long‐term health. Clear communication of health check results, using simple language and offering follow‐up support, would help individuals better understand their results and what actions to take.

Addressing psychological barriers by providing more information upfront about what to expect, along with reassurance that early detection leads to better outcomes, could reduce anxiety about attending. Building trust in marginalised communities by involving respected local leaders and healthcare professionals could further encourage participation.

Improving accessibility and flexibility is also vital. Offering appointments in the evenings or on weekends and holding health checks in community spaces like local hubs or libraries, would make it easier for people to attend. Mobile clinics could also reach more remote or underserved areas.

Digital tools can play a key role in engagement by simplifying the booking process and providing follow‐up advice after checks. Social media and online platforms should be used more effectively to spread awareness about the benefits of NHS Health Checks.

Lastly, policymakers should consider shifting from the current activity‐based payment system to one that focuses on outcomes, ensuring better care and follow‐up. Stronger collaboration between local authorities, healthcare providers and community organisations is essential, especially in areas where trust in healthcare is low. Local figures such as community leaders could help build this trust and encourage more people to take part in the programme.

### 4.5 Strengths and Limitations

One of the key strengths of this study is its use of participatory research. Employing PRAs from the communities being studied enhanced the data by ensuring that the voices of those most affected by the programme were central to the research process. This helped to reduce power imbalances that can sometimes exist between participants and researchers, further enhancing the authenticity of the data collected. The participatory approach ensured that the experiences and perspectives shared were reflective of the actual concerns of individuals in socioeconomically deprived areas, adding credibility to the findings.

However, several limitations must be acknowledged. While the relatively small sample size (*n* = 17) may limit the extent to which findings can be generalised to other regions or demographic groups, this was not the primary aim of the study. Rather, the focus was on exploring barriers and facilitators within a specific geographical and socioeconomic context in North East England. As such, the sample size was sufficient to address the research questions and provided rich and meaningful insights into the challenges faced by this population. Furthermore, thematic analysis does not require large sample sizes to yield rich and meaningful data. Braun and Clarke [40] suggest that smaller samples are appropriate for this type of analysis, as the focus is on depth and complexity of understanding rather than representativeness. Additionally, it has been found that data saturation often occurs within the first 12 interviews [41].

While this study primarily explored barriers and facilitators to NHS Health Check uptake in socioeconomically deprived areas, it is important to acknowledge the underrepresentation of ethnic minority groups in our sample. Despite extensive recruitment efforts through community organisations and networks, only one participant identified as Black African, which limits the ability to draw conclusions about the unique experiences and barriers faced by ethnic minority populations. This underrepresentation may reflect the demographic composition of the North East of England, where ethnic minorities account for a smaller proportion of the population compared to other regions [42]. However, the lower participation rates among ethnic minorities in preventive health services, as highlighted in previous research, emphasise the need for targeted approaches to engage these communities.

Furthermore, the study did not include NHS employees (GPs and other healthcare staff), due to time constraints impacting the ability to obtain the necessary additional ethics approval. This is a limitation as healthcare professionals are directly involved in delivering NHS Health Checks and could have provided useful insights into operational challenges, patient engagement strategies and the practicalities of implementing the programme. Their perspectives may have highlighted gaps between policy‐level decision‐making and frontline delivery. However, the perspectives of the stakeholders recruited for the present study were still highly relevant, as they were involved in policymaking and strategic decision‐making related to the NHS Health Check programme. Their insights into barriers, facilitators and potential improvements were valuable for understanding the broader challenges and opportunities in increasing uptake in underserved communities.

### 4.6 Future Directions

Future research should aim to ensure greater representation of ethnic minority groups, who are often disproportionately affected by health disparities, to better understand and address the intersection of race, ethnicity and socioeconomic deprivation in accessing health checks. Additionally, research should explore the potential of digital health interventions in increasing engagement with NHS Health Checks, as recommended by stakeholders in the present study.

Furthermore, future research could build on the present findings by including healthcare professionals, such as GPs and practice nurses in North East England, to provide a more comprehensive view of how the programme is delivered in clinical settings and how it can be enhanced from both a policy and frontline healthcare perspective.

## 5 Conclusion

NHS Health Checks have significant potential in preventive healthcare, but this study highlights persistent barriers faced by individuals in socioeconomically deprived communities in the North East of England. These barriers included low awareness, fear of health issues, mistrust in healthcare professionals, and difficulties accessing appointments. However, participants expressed a willingness to engage if communication and access were improved. Stakeholders also noted structural challenges, such as underfunding and the need for better collaboration between healthcare providers, local authorities, and community organisations. They suggested that digital tools and offering health checks in more accessible community locations could help increase participation.

Addressing these barriers, including limited cultural health capital and structural inequities, alongside implementing targeted communication strategies and community‐based interventions, could enhance the effectiveness of the NHS Health Check programme. This approach holds promise for reducing health inequalities and improving long‐term outcomes in underserved communities.

## Author Contributions

**Judith Eberhardt:** conceptualisation, investigation, funding acquisition, writing – original draft, methodology, validation, writing – review and editing, formal analysis, project administration, supervision, data curation. **Laura Kane:** investigation, validation, writing – review and editing, writing – original draft. **Robert Portman:** formal analysis, writing – original draft, writing – review and editing. **Jonathan Ling:** conceptualisation, funding acquisition, writing – review and editing, writing – original draft. **Tracy Goddard:** validation, writing – review and editing, investigation. **Mark Johnston:** validation, writing – review and editing, investigation. **Claire Robinson:** funding acquisition, writing – review and editing, conceptualisation. **Abigail Reay:** validation, writing – review and editing. **Andrew Divers:** writing – review and editing. **Dorothy Newbury‐Birch:** writing – review and editing, funding acquisition, conceptualisation.

## Conflicts of Interest

The authors declare no conflicts of interest.

## Supporting information

Supporting information.

## Data Availability

The data supporting the findings of this study are available upon reasonable request from the corresponding author. The data are not publicly available due to privacy or ethical restrictions.
