# Supplementary material for: Barriers and Facilitators of NHS Health Checks in Socioeconomically Deprived Communities in the North East of England: A Qualitative Study With Peer Researchers
Source: Health Expect. 2025 Apr 15;28(2):e70199. doi: 10.1111/hex.70199 (PMC12000538; doi:10.1111/hex.70199)
Supplement: Supplementary file 1 — Supporting information. [file HEX-28-e70199-s001.docx]

Table S1. Interview Schedule for Community Members

| **Topic** | **Questions** |
| --- | --- |
| Awareness and Perception | Did you know about the NHS Health Check programme before today? If yes, how did you find out about it?   - Prompt: Do you remember when or where you first heard about it? - Probe: Did that information make you want to learn more or do something about it?   What do you think an NHS Health Check does?   - Prompt: Could you tell me what you think happens during a health check? - Probe: Where do you think this knowledge came from? Friends, family, media? |
| Personal Experience | Have you ever been invited to have an NHS Health Check? If yes, can you tell me about what happened, from the time you were invited?   - Prompt: What were your first thoughts when you got the invitation? - Probe: If you decided not to go, can you tell me why?   If you have never been invited, would you be interested in having a health check? Why or why not?   - Prompt: What would make you feel that a health check is a good thing to do? - Probe: Do you have any worries or questions about going for a health check? |
| Barriers to Participation | Why do you think some people might not go for their NHS Health Check, especially in your community or area?   - Prompt: Have you heard any friends or family members talk about why they haven't gone? - Probe: Do you think there's enough information or support to make people want to go?   What might stop you or others from going to a health check?   - Prompt: Can you think of a time when this *[i.e., the barrier preventing someone from going for their health check]* came up for you or someone you know? - Probe: How do you think this could be tackled? |
| Suggestions for Improvement | How could NHS Health Checks be made easier to get, or more attractive to people in your community?   - Prompt: What services or programmes are popular in your community? Why do you think they work? - Probe: How could we use what works there with the NHS Health Checks?   Are there any specific changes to the way information about NHS Health Checks is given to people that you think could help?   - Prompt: Have you ever received health information that you found really clear and helpful? What made it stand out? - Probe: How can we use what works in those programmes to talk about NHS Health Checks? |
| Final Thoughts | Is there anything else you'd like to share about your thoughts or experiences related to NHS Health Checks?   - Prompt: Is there a story or experience you'd like to share that we haven't talked about yet? - Probe: What change would you most like to see, from what we’ve talked about today? |

Table S2. Interview schedule for stakeholders

| **Topic** | **Questions** |
| --- | --- |
| Background and Role | Can you describe your role and responsibilities, or your professional interests in relation to the NHS Health Check programme? |
| Programme Insights | In your view, how does the NHS Health Check programme contribute to broader public health goals, especially in terms of preventive healthcare?   - Prompt: Can you share an example where an early detected condition through the health check led to a successful intervention? - Probe: How do you think this programme shifts the focus from treatment to prevention in public health?   What are some unique challenges you've encountered in promoting the NHS Health Check programme within deprived communities, and how have these challenges evolved over time?   - Prompt: Could you describe a situation where a typical challenge was overcome? - Probe: Have any recent societal or technological changes impacted these challenges? |
| Engagement and Accessibility | Could you detail any specific outreach or communication strategies that have been tailored for engaging with deprived communities? How were these strategies developed?   - Prompt: What was the inspiration or basis for one of your more successful strategies? - Probe: How do you assess and iterate on these strategies based on community response?   Thinking about the effectiveness of current engagement strategies, are there any particular insights or feedback from the community that have informed your thinking about potential improvements?   - Prompt: Is there a piece of feedback that really made you think differently about how you engage? - Probe: How has this feedback been translated into action or changes in strategy? |
| Barriers and Facilitators | Beyond individual-level barriers, are there any systemic or structural issues that you believe contribute to lower uptake rates of NHS Health Checks in certain areas?   - Prompt: Can you give an example of a structural issue and how it impacts uptake? - Probe: What steps can be taken to mitigate such systemic issues?   Have there been any notable initiatives or collaborations that helped with the uptake of NHS Health Checks in deprived areas? What do you think made these initiatives successful?   - Prompt: Could you tell us about one initiative that stands out to you? - Probe: What elements of this initiative do you think could be applied more broadly? |
| Programme Improvement | Considering the changing healthcare landscape, how do you envision the NHS Health Check programme evolving to meet the needs of diverse communities, especially those that are underserved?   - Prompt: Are there emerging trends or technologies you see as key to the future of the programme? - Probe: How can the programme be more adaptive to the specific needs of different communities?   From a policy or operational perspective, what are some key changes you feel are necessary to enhance the effectiveness and reach of the NHS Health Check programme?   - Prompt: If you could make one significant change to the programme, what would it be? - Probe: How would this change address current gaps in programme delivery? |
| Closing | Reflecting on our discussion, are there any critical areas or innovative ideas you think need further exploration to optimise the implementation of the NHS Health Check programme in deprived areas?   - Prompt: Is there an idea or concept we haven't discussed that you believe deserves more attention? - Probe: How could this idea be tested or implemented in a practical way? |
